# Mitochondrial polymorphism of sea beet (*Beta vulgaris* ssp. *maritima*), a species with cytoplasmic male sterility

**DOI:** 10.1371/journal.pone.0332940

**Published:** 2025-09-23

**Authors:** Tomoki Murata, Hiroyo Kagami-Katsuyama, Mion Oishi, Haruto Tanaka, Chihiro Sano, Jun Kashikura, Ryo Hayakawa, Keishi Kubota, Eigo Taniguchi, Keita Suzuki, Kazuyoshi Kitazaki, Tomohiko Kubo

**Affiliations:** 1 Research Faculty of Agriculture, Hokkaido University, Sapporo, Japan; 2 Department of Medical Management and Informatics, Hokkaido Information University, Ebetsu, Japan; Al Muthanna University, IRAQ

## Abstract

Cytoplasmic male sterility (CMS), a form of mitochondrion-induced male sterility, is a valuable trait for hybrid breeding in crop production. However, CMS is not universally present across all crop species, prompting ongoing efforts to identify CMS sources within genetic resources. The discovery of CMS could be facilitated by the development of predictive indices indicating the presence of CMS-associated mitochondria. One theory has proposed that populations containing CMS-expressing plants exhibit elevated mitochondrial polymorphism. Sea beet, a species known to harbor CMS, provides a suitable system for testing this prediction. We first conducted network analysis by using mitochondrial single nucleotide polymorphism sites extracted from publicly available nucleotide sequence data. The resulting network, constructed from 270 sea beet sequences, was complex yet characterized by several star-like clusters. Haplotypes from Mediterranean region were dispersed across the network, while those from Atlantic coast tended to cluster, supporting the hypothesis that sea beet originated in the Mediterranean area and later migrated to the Atlantic coast. We then analyzed four mitochondrial minisatellites—variable number of tandem repeat loci— across 973 plants of 172 sea beet accessions. Combination of alleles at these four loci defined 29 distinct mitotypes. Analysis of mitotype distribution revealed similarly high mitochondrial polymorphism (~0.87) in both the Mediterranean and the Atlantic regions, despite their genic differentiation. Most subregions within these areas contained 8–15 mitotypes; however, Denmark was a notable exception, with only a single mitotype detected among 139 plants from 11 accessions. This mitochondrial monomorphism contrasts with the diversity observed in neighboring Atlantic subregions, indicating that Danish sea beet constitutes an exceptional population. Notably, the Danish mitotype was not associated with any CMS, suggesting that the population may be devoid of CMS mitochondria— a condition in which the proposed mechanism for increased mitochondrial polymorphism would not be operative.

## Introduction

Mitochondria play important roles in various cellular processes beyond ATP production, including signal transduction and the regulation of programmed cell death [1,2]. Mutations in mitochondrial DNA (mtDNA), despite the genome’s limited genes content, can give rise to a broad spectrum of phenotypes. In plants, mitochondrial defects frequently impair male reproductive development, resulting in cytoplasmic male sterility (CMS) [3]. CMS is a valuable trait in agriculture, which enables the use of ideal female plants for hybrid seed production (i.e., hermaphroditic plants lacking male function can effectively serve as female) [4]. However, CMS has not been identified in all crop species, and efforts to discover suitable CMS mitochondria are ongoing (e.g., [5]). Developing an index to predict the presence of CMS-associated mitochondria in genetic resources would greatly facilitate their identification.

One theory proposes a mechanism by which populations maintain plants expressing CMS: because male sterility is inherently disadvantageous (resulting in the absence of pollen), CMS-associated mitochondria are thought to compensate by conferring a selective advantage [6]. Conversely, nuclear suppressors can restore fertility by suppressing CMS, but the theory assumes that the restoring alleles are disadvantageous in plants harboring non-CMS mitochondria [7]. Given the pleiotropic effects of CMS mitochondria and the restoring alleles of nuclear suppressors, the theory predicts balancing selection between CMS and non-CMS mitochondria, as well as among nuclear suppressor alleles, thereby maintaining male-sterile plant within the population [7]. A key implication of this theory is the accumulation of mitochondrial variants in the population, leading to high mitochondrial polymorphism [8].

Mitochondrial polymorphism has attracted considerable attention and has been widely utilized in evolutionary biology studies in animal [1]. In contrast, instances of mitochondrial polymorphism in plants are relatively rare (plastid DNA polymorphism are used instead). This is attributed to the larger size of the plant mtDNA, the presence of homologous sequences shared with nuclear and plastid DNAs, and a lower copy number of mtDNA molecule per cell [3,9–11], as well as a much slower nucleotide substitution rate compared to animal mtDNA [12]. Therefore, plant mitochondrial polymorphism should be assessed with these features in mind. Advances in high-throughput sequencing technologies, including next-generation sequencing and its successors, have made plant mtDNA easier to analyze. However, the presence of homologous sequences to nuclear- and plastid DNA within plant mtDNA remains problematic [11], and careful data processing is still required.

Mitochondrial polymorphism can be assessed by analyzing highly variable regions. In animal mitochondria, a non-coding region, known as the D-loop, accumulates nucleotide sequence variation and has been widely studied to investigate mitochondrial polymorphism [1]. In contrast, no common non-coding region with sufficient polymorphism for evolutionary studies has been identified in plant mitochondria. However, plant mitochondria possess loci with variable numbers of tandem repeats, such as simple sequence repeats and minisatellites [13,14]. Haplotype diversity can be defined by the allelic combinations at these loci, and mitochondrial polymorphism is evaluated based on haplotypes variation (e.g., [15]).

*Beta* *vulgaris* includes cultivar groups such as sugar beet and garden beet (both classified as *B. vulgaris.* subsp. *vulgaris*). Hybrid breeding using CMS is conducted in both groups [16,17], making CMS an important trait in breeding programs. In *B. vulgaris*, three CMS-associated mitochondrial types have been identified to date [18], one of which is widely used in practical breeding, while the others are considered potential alternatives [19].

The three CMS-associated mitochondrial types were identified in sea beet (*B. vulgaris* ssp. *maritima*) [18], a wild relative of cultivated beets. Sea beet predominantly inhabits coastal regions of Europe, North Africa, Asia Minor, and the Near East [20]. Its origin is thought to be the Mediterranean region with subsequent spread to the Atlantic coast following the end of the last ice age [20,21]. Sea beet individuals carrying the three CMS-associated mitochondria have been found in both the Mediterranean region and the Atlantic coast [22,23], suggesting that these CMS evolved in the Mediterranean and later migrated into the Atlantic; However, the reverse migration pattern cannot be ruled out.

Sea beet serves as an important genetic resource for sugar beet and garden beet breeding [24]. While the nuclear polymorphism of sea beet has been extensively studied (e.g., [25–28]), its mitochondrial polymorphism has received comparatively less attention. Given the prediction that populations containing CMS plants may exhibit increased mitochondrial polymorphism, the mitochondrial diversity of sea beet represents an interesting subject of study. In this context, we sought to investigate the extent of mitochondrial polymorphism in sea beet. In this study, we performed network analysis based on single nucleotide polymorphisms (SNPs) of sea beet mtDNA to explore whether the mitochondrial evolutionary pattern aligns with the proposed dissemination route of sea beet. Next, we analyzed mitochondrial minisatellite loci of sea beet to examine the macroscopic polymorphic patterns. Our results suggest that the dissemination of sea beet mitochondria is a complex process, generally following a Mediterranean-to-Atlantic direction. During this process, novel mitochondrial variants have evolved and been maintained, preserving mitochondrial polymorphism. Notably, while 8–15 mitotypes were observed across most subregions, Danish accessions exhibited mitochondrial monomorphism and, intriguingly, they were free of CMS.

## Materials and methods

### Network analysis of mitochondrial sequences

Kubota et al. [23] identified 749 mitochondrial SNPs from 600 *B. vulgaris* plants. From this dataset we excluded the data of non-sea beet individuals, such as *B. v.* ssp. *vulgaris*. The remaining 270 sea beet samples span the Atlantic coast of Europe and the Mediterranean region. Based on the nucleotides at these SNPs, we constructed a 749-bp mitochondrial sequence for each of the 270 sea beet individuals. These sequences are provided as a FASTA file (S1 Appendix). The haplotype network was constructed using the *Pegas* R package [29], with the TCS method applied for network construction. The network was then manually visualized.

### Plant materials

**Fig 1:**
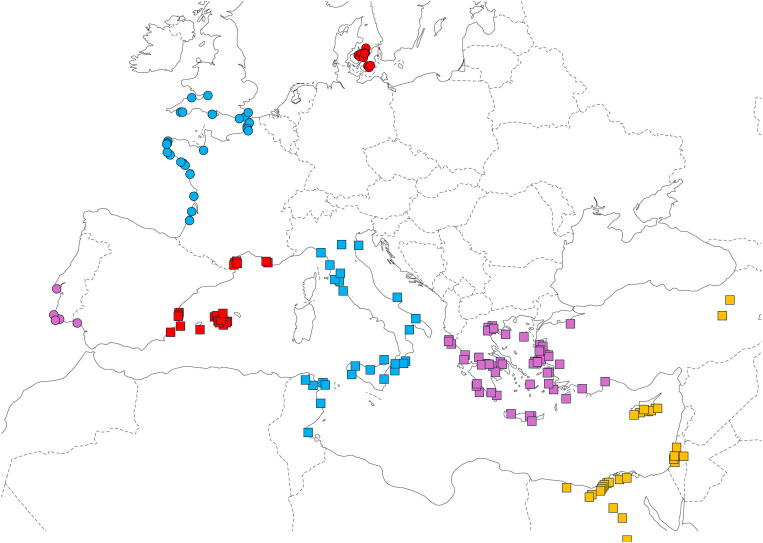
Collection sites of sea beet accessions used for mitotype analysis. Red circles: Denmark (DNK); blue circles: Atlantic coast of France and the United Kingdom (Atl:FRA-GBR); purple circles: Atlantic coast of Portugal and Spain (Atl:POR-ESP); red squares: Mediterranean coast of Spain and France (Med:ESP-FRA); blue squares: Mediterranean coast of Tunisia and Italy (Med:TUN-ITA); purple squares: Mediterranean coast of Greece and Turkey (Med:GRC-TUR); yellow squares: Mediterranean coast and inland of Turkey, Cyprus, Israel and Egypt (two accessions collected from the upstream region of the Nile river are not shown on the map) (Med:TUR-CYP-ISR-EGY).

Sea beet genetic resources for minisatellite analysis were obtained from the Nordic Genetic Resources Center (NordGen), the Leibniz Institute of Plant Genetics and Crop Plant Research (IPK), and the United States Department of Agriculture (USDA) (S1 Table). The collection sites of these resources are shown in Fig 1. These are *ex situ* propagated germplasm collections. Seeds were sown in a greenhouse, and the resulting plantlets were subsequently transplanted in the field to grow.

### DNA marker analysis

Total cellular DNA was isolated according to the procedure described in [30]. Four mitochondrial minisatellite loci (TR1 to TR4) in sea beet were PCR amplified according to [31]. PCR products were electrophoresed in an agarose gel alongside markers whose number of repeat units were determined by nucleotide sequencing. Any ambiguity in repeat numbers was resolved by direct sequencing of the PCR products using an ABI3130 Genetic Analyzer (Thermo Fisher Scientific, Waltham, MA, USA). Mitochondrial types (mitotypes) were defined by the combination of alleles at the four loci and were named according to [32–34]. Novel combinations of alleles were assigned new names and these mitotypes are summarized in S2 Table. The dataset is available in S3 Table. PCR detection of *orf129* was carried out according to [33], using the oligonucleotide primers 5′-ATCCATGGTGATGAATCCTTATATTCTGC-3′ and 5′-CTAGAGCTCTCACTGTGAGAGATAG-3′. The resulting amplicon (expected size: 0.4 kbp) was electrophoresed on a 2%agarose gel.

### Mitotype polymorphism analysis

Mitotype polymorphism was summarized by calculating the diversity index *H* [15], using the following equation:


$$H=\text{1}-\sum {p_{i}}^{\text{2}}$$

where *p*_*i*_ denotes frequency of the *i*-th mitotype. Prior to calculation in Microsoft Excel (Microsoft Japan, Tokyo, Japan), singletons were removed [35]. Genic differentiation was calculated using GenePop [36] with default parameter.

## Results

### Haplotype network of sea beet mitochondria

**Fig 2:**
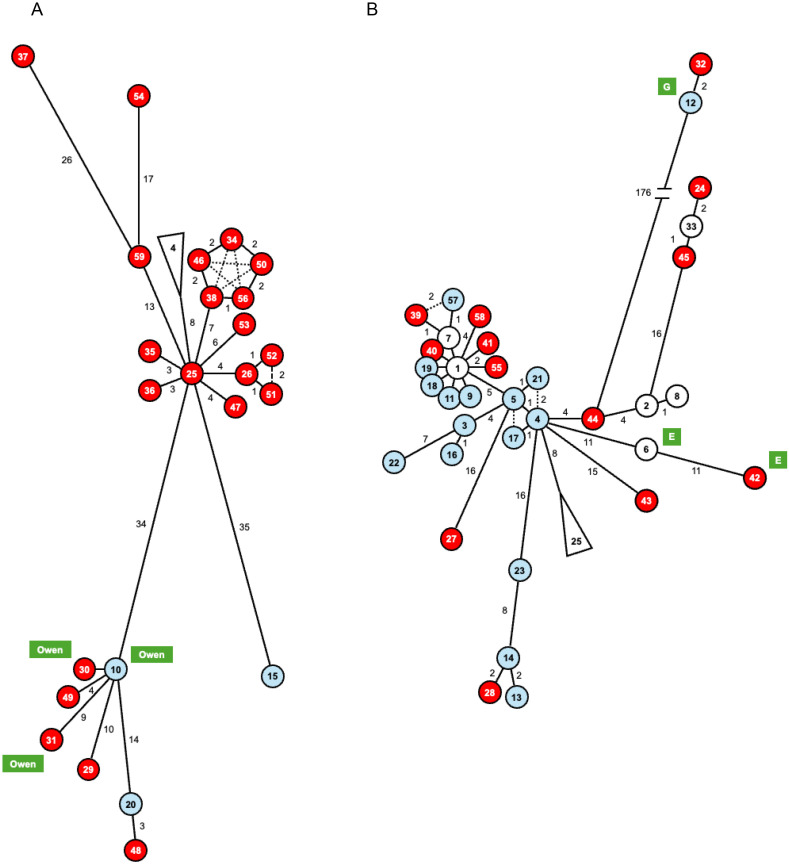
Haplotype network of sea beet mitochondria. Circles represent mitochondrial haplotypes. Dashed lines indicate alternative connections. Branch length is generally proportional to the number of mutations between the haplotypes, except for those among Haplotypes 34, 50, 56, 38 and 46 which are too similar to resolve accurately, and for the branch between Haplotypes 44 and 12, where the number of mutations is too large to depict to scale. Alternative branches among Haplotypes of 1, 7, 9, 11, 18, 19, 39, 40, and 57 are not shown. The original network is split at Haplotypes 25 and 4 (denoted by triangles) into Subnetwork A (panel A) and Subnetwork B (panel B). Accessions details for each haplotype are provided in S4 Table. Colors of circles indicate the collection sites of the accessions: red for Mediterranean, sky blue for Atlantic, and white for both. Haplotypes associated with accessions carrying Owen-type, G-type, or E-type CMS are shown with green highlights.

To investigate the relationship between mitochondrial genealogy and collection sites, the 270 sequences from sea beets that were collected from the Atlantic coast of Europe or the Mediterranean region were subjected to haplotype network analysis. The resulting network was highly complex, leading us to divide it into two parts, designated Subnetwork A and Subnetwork B (Fig 2, with larger image provided in S1 Fig).

The network contains 59 haplotypes, designated Haplotype 1 to Haplotype 59. Since each accession is represented by a single plant, we considered it inappropriate to evaluate haplotype frequency (accessions corresponding to each haplotype are listed in S4 Table). The Subnetwork A exhibits a star-like structure comprising 24 haplotypes, with Haplotype 25 positioned at the center. Several branches, such as those connecting Haplotypes 25 to Haplotype 15, Haplotypes 25 to Haplotypes 10, and Haplotypes 59 to Haplotypes 37, are notably longer than others. By mapping the collection sites to the haplotypes, we found that 21 of the 24 haplotypes in Subnetwork A were collected from the Mediterranean region (Fig 2A). The remaining three haplotypes in Subnetwork A were collected from the Atlantic coast. Subnetwork B contains 35 haplotypes and exhibits a more complex structure compared to Subnetwork A. As is the case of Subnetwork A, some branches in Subnetwork B are notably longer, such as the branch between Haplotype 44 and Haplotype 12. The collection sites associated with Subnetwork B haplotypes include the Atlantic coast (16 haplotypes), the Mediterranean area (13 haplotypes), and both regions (6 haplotypes). Overall, haplotypes from the Atlantic coast are concentrated in Subnetwork B (16 of the 19 haplotypes). In contrast, while Mediterranean haplotypes predominate in Subnetwork A, in Subnetwork B the number of Mediterranean haplotypes is comparable to that of Atlantic haplotypes (13 Mediterranean haplotypes vs. 16 Atlantic haplotypes).

### Mitochondrial polymorphism is generally high in both the Atlantic coast and Mediterranean area

We next investigated the minisatellite polymorphism of sea beet mitochondria. A total of 973 plants (172 accessions) (S1 Table) were analyzed. Based on previous studies of sea beet nuclear genome polymorphism [25], the collection sites of the accessions were categorized as follows: Denmark (DNK), Atlantic coast of France and the United Kingdom (Atl:FRA-GBR), Atlantic coast of Portugal and Spain (Atl:PRT-ESP), Mediterranean coast of Spain and France (Med:ESP-FRA), Mediterranean coast of Tunisia and Italy (Med:TUN-ITA), Mediterranean coast of Greece and Turkey (Med:GRC-TUR), and Mediterranean coast and inland regions of Turkey, Cyprus, Israel and Egypt (Med:TUR-CYP-ISR-EGY) (Fig 1).

We determined the alleles of four mitochondrial minisatellite loci in sea beet, designated TR1, TR2, TR3, and TR4 (S3 Table). Across the 973 plants analyzed, the number of alleles per locus ranged from three to ten (S3 Table). The locus with the highest allelic diversity was TR1, which is located within recombination-active repeated sequences in the *B. vulgaris* mitochondrial genome [31]. Mitotypes were defined based on the combination of alleles at the four minisatellite loci (S2 Table), resulting in the identification of 29 distinct mitotypes, such as min01, min03, min04 and min06. Some mitotypes reported in previous studies [32] were not found in this study, and therefore certain numbers were skipped (Tables 1 and 2).

**Table 1:** Mitotype polymorphism of sea beet.

Area ^*1^ and subregion	No. of accessions	No. of plants	No. of mitotypes	No. of private mitotype	Diversity index (H)
DNK	11	139	1	0	0
Atl total (without DNK)	31	292	17	4	0.8725
Atl:FRA-GBR	25	244	13	3	0.8370
Atl:POR-ESP	6	48	8	1	0.8299
Med total	130	542	24	7	0.8733
Med:ESP-FRA	21	87	9	1	0.6930
Med:TUN-ITA	26	113	15	4	0.8383
Med:GRC-TUR	54	234	13	0	0.8424
Med:TUR-CYP-ISR-EGY	29	108	13	1	0.7778
Total	172	973	29	10	0.8656

*^1^ DNK, Denmark; Atl:FRA-GBR, Atlantic coast of France and the United Kingdom; Atl:POR-ESP, Atlantic coast of Portugal and Spain; Med:ESP-FRA, Mediterranean coast of Spain and France; Med:TUN-ITA, Mediterranean coast of Tunisia and Italy; Med:GRC-TUR, Mediterranean coast of Greece and Turkey; and Med:TUR-CYP-ISR-EGY, Mediterranean coast and inland areas of Turkey, Cyprus, Israel and Egypt.

**Table 2:** Mitotypes and number of sea beet plants in areas and subregions.

Area and subregion	Mitotype																												
	min01	min03	min04	min06	min07	min08	min09	min10	min11	min15	min16	min17	min18	min19	min20	min23	min24	min26	min27	min32	min33	min34	min35	min36	min37	min38	min39	min40	min41	Total
DNK	0	0	0	0	139	0	0	0	0	0	0	0	0	0	0	0	0	0	0	0	0	0	0	0	0	0	0	0	0	139
Atl:FRA-GBR	5	0	4	8	56	5	59	0	29	5	0	0	26	0	0	0	1	3	37	6	0	0	0	0	0	0	0	0	0	244
Atl:POR-ESP	0	0	0	2	4	0	0	0	4	6	0	0	0	0	12	0	0	0	0	0	0	12	4	4	0	0	0	0	0	48
Med:ESP-FRA	0	0	2	0	4	0	43	0	17	0	0	3	12	2	3	1	0	0	0	0	0	0	0	0	0	0	0	0	0	87
Med:TUN-ITA	0	1	0	5	9	0	11	1	35	13	1	7	19	4	4	0	0	0	0	0	0	0	0	1	1	1	0	0	0	113
Med:GRC-TUR	0	0	0	48	28	3	18	18	3	31	0	1	61	5	0	0	0	0	0	0	12	0	1	0	0	0	5	0	0	234
Med:TUR-CYP-ISR-EGY	0	0	0	44	10	1	12	0	0	1	0	0	18	0	0	0	0	3	5	0	1	0	2	0	0	0	5	5	1	108
Total	5	1	6	107	250	9	143	19	88	56	1	11	136	11	19	1	1	6	42	6	13	12	7	5	1	1	10	5	1	973
Atl total (w/o DNK)	5	0	4	10	60	5	59	0	33	11	0	0	26	0	12	0	1	3	37	6	0	12	4	4	0	0	0	0	0	292
Med total	0	1	2	97	51	4	84	19	55	45	1	11	110	11	7	1	0	3	5	0	13	0	3	1	1	1	10	5	1	542
Total (w/o DNK)	5	1	6	107	111	9	143	19	88	56	1	11	136	11	19	1	1	6	42	6	13	12	7	5	1	1	10	5	1	834

Table 1 indicates that only a single mitotype (min07) was detected among 139 plants representing 11 accessions collected in DNK. To assess whether this mitochondrial monomorphism is consistent across datasets, we examined the presence of DNK accessions in the haplotype network (S4 Table). In the nucleotide sequence dataset used for network analysis, 18 sequences were derived from DNK. All 18 sequences were identical and belonged to Haplotype 1 (S4 Table and Fig 2B). Notably, the 18 DNK sequences originated from accessions preserved in the USDA collection, whereas the 11 DNK accessions for our minisatellite analysis were obtained from NordGen. These findings suggest that mitochondrial polymorphism in DNK sea beet populations is markedly reduced. Thus, it may be appropriate to treat DNK accessions as a distinct population in terms of mitochondrial polymorphism.

Excluding the DNK accessions, the most frequent mitotype among the remaining 834 plants was min18, accounting for approximately ~15% of the samples (Table 2). The combined frequency of the four most common mitotypes (min06, min07, min09, and min18) exceeded ~53%. Among all mitotypes, min07 was the only one identified across all geographic areas and subregions (Table 2). In addition to min07, the more common mitotypes were min06, min09, min11, min15 and min18 (Table 2). Apart from min06, they only differ at the TR1 locus (S2 Table). Each additional group – Atl:FRA-GBR, Atl:POR-ESP, Med:ESP-FRA, Med:TUN-ITA, Med:GRC-TUR and Med:TUR-CYP-ISR-EGY – harbored between 8 and 15 mitotypes (Table 2).

We summarized mitotype polymorphism across all sea beet samples analyzed, obtaining a diversity index of 0.8656 (Table 1). Sea beet populations are known to be subdivided into Atlantic and Mediterranean types [37]. Given that the DNK accessions were mitochondrially monomorphic (see above), we treated them as a different group. The mitotype polymorphism indices for the DNK, Atlantic (excluding DNK), and Mediterranean groups were 0, 0.8725, and 0.8733, respectively (Table 1). Genic differentiation between each pair of these three populations was significant (*p* < 0.001) (Table 3).

**Table 3:** Genic differentiation between pairs of three populations.

Population pair	χ^2^ value	d.f.	*p* value
DNK vs. Atl (w/o DNK)	>52.20776	6	<1.69e-09
DNK vs. Med	>76.98012	8	<1.98e-13
Atl (w/o DNK) vs. Med	>89.33081	8	<6.36e-16

We also examined mitotype polymorphism within the subregions of the Atlantic and Mediterranean area (Table 1). The diversity indices for Atl:FRA-GBR and Atl:POR-ESP were 0.8370 and 0.8299, respectively. In the Mediterranean subregions, the values were 0.6930 for Med:ESP-FRA, 0.8383 for Med:TUN-ITA, 0.8424 for Med:GRC-TUR and 0.7778 for Med:TUR-CYP-ISR-EGY. These results indicate that the extreme decrease in mitotype polymorphism is unique to the DNK group.

### Frequency of CMS mitochondria in sea beet accessions

The three CMS-associated mitochondrial types in *B. vulgaris* have been tightly linked to specific mitotypes: G-type, Owen-type, and E-type, corresponding to min01, min04, and min06, respectively [32,33,38]. In the present study, min01 was detected in one accession from Atl:FRA-GBR and min04 was identified in accessions from Atl:FRA-GBR and Med:ESP-FRA (Table 2 and S1 Table). In contrast, although E-type CMS mitochondria are associated with min06, not all min06 mitotypes are E-type CMS [32]. We examined whether the min06 sea beet plants identified in this study harbored the mitochondrial gene *orf129*, a unique ORF associated with E-type CMS mitochondria [39]. All min06 plants were subjected to PCR amplification of *orf129*. PCR-positive results were obtained for all eight plants from Atl:FRA-GBR, the two plants from Atl:POR-ESP, all five plants from Med:TUN-ITA, and five plants (two accessions) from Med:GRC-TUR, whereas the remaining min06 plants were PCR-negative (Table 4). Thus, among the 973 plants analyzed, five plants carried G-type CMS mitochondria, six carried Owen-type CMS mitochondria and 20 plants carried E-type CMS mitochondria, corresponding to an overall CMS mitochondrial frequency of 3.1%. We may underestimate the frequency of E-type CMS mitochondria due to potential false negatives, since our detection system depends on PCR amplification of *orf129* from the min06 plant. In comparison, a careful inspection of the data reported by Kubota et al. [23] indicated CMS mitochondria frequency is 7.8%. Based on these results, the frequency of CMS mitochondria in sea beet can be estimated to range from approximately 3–8%.

**Table 4:** Number of sea beet plants with or without *orf129* among individuals carrying the mitochondrial mitotype min06.

Subregion	Accession	Origin	*orf129*	
			+	–
Atl:FRA-GBR	BETA 1127	FRA	8	0
Atl:POR-ESP	BETA 1276	PRT	2	0
Med:TUN-ITA	BETA 3824	ITA	5	0
Med:GRC-TUR	BETA 1017	TUR	0	2
	BETA 1074	TUR	0	5
	BETA 1079	TUR	0	1
	BETA 1113	GRC	0	5
	BETA 1287	TUR	0	1
	BETA 1415	GRC	0	3
	BETA 963	GRC	2	1
	BETA 973	GRC	0	5
	PI 546437	GRC	0	5
	PI 546518	GRC	0	3
	PI 546520	GRC	0	6
	PI 604508	GRC	0	3
	PI 604517	GRC	0	3
	W6 21681	GRC	3	0
Med:TUR-CYP-ISR-EGY	BETA 1227	ISR	0	4
	BETA 1260	CYP	0	5
	BETA 1565	ISR	0	2
	BETA 1844	CYP	0	1
	BETA 1853	CYP	0	2
	BETA 980	ISR	0	4
	BETA 474	TUR	0	4
	PI 562579	EGY	0	5
	PI 562581	EGY	0	3
	PI 562585	EGY	0	1
	PI 562586	EGY	0	2
	PI 562587	EGY	0	4
	PI 562589	EGY	0	3
	PI 562590	EGY	0	1
	PI 562596	EGY	0	2
	PI 562601	EGY	0	1
Total			20	87

## Discussion

We investigated mitochondrial polymorphism in sea beet using both SNPs and minisatellites. The mitochondrial SNPs identified by Kubota et al. [23] enabled us to construct a haplotype network for sea beet. The resulting network is highly complex and contains several lineages with longer branches than others. Because our sampling may not have captured the full extent of mitochondrial diversity in sea beet, it is possible that these longer branches could be solved with more complete sampling. Alternatively, the longer branches might represent ancient polymorphisms, where certain haplotypes have persisted over longer evolutionary timescales either by chance or by selective pressure. Another possibility is that nucleotide substitution rate in sea beet has fluctuated over time. Although the nucleotide substitution rate of plant mitochondria is very low, considerable variation among plant species has been reported, and species with unusually high rate have been identified [40–43]. If the mechanism controlling the substitution rate (e.g., [44]) were temporally impaired during sea beet evolution, it could have led to a transient acceleration of mutations, resulting in the accumulation of changes over a relatively short evolutionarily period and, consequently, the appearances of longer branches in the network. This possibility has been proposed previously [7].

According to Kubota et al. [23], some accessions with the three CMS mitochondrial types were included in the dataset used for haplotype network analysis. It is interesting to note that haplotypes of these CMS-bearing accessions are located at or near to the tips of the longer branches in the network: Haplotypes 10, 30 and 31 include accessions with Owen-type CMS mitochondria (Fig 2), while those with G-type and E-type CMS mitochondria are found in Haplotype 12 and haplotypes 6 and 42, respectively (Fig 2). A similar association between CMS and accelerated mitochondrial evolution has been reported in a freshwater snail [45]. However, it remains unknown whether all the haplotypes at the tips of longer branches induce male sterility – for example Haplotype 37 in Subnetwork A (Fig 2) has no known CMS association. Investigating the relationship between mitochondrial evolutionary rate and CMS remains an important area for future research in both plant and animal.

The haplotype network of sea beet mitochondria appears as a combination of several star-like structures, with no evident loop structure, except among clusters of highly similar haplotypes (Fig 2). This topology indicates that sea beet mitochondria have evolved through simple mutational accumulation with no detectable role for recombination. Since mitochondria in *B. vulgaris* are maternally inherited, this observation is consistent with expectations. However, recombination cannot be entirely ruled out, as certain nucleotide sequence stretches are shared by two distinct clades in the mitochondrial phylogenetic tree of *B. vulgaris* mitochondria [23,46].

In the mitochondrial haplotype network, haplotypes found in Mediterranean accessions but not in Atlantic ones (referred to as Mediterranean haplotypes) are scattered throughout the network. In contrast, Atlantic haplotypes are concentrated in Subnetwork B and are rare in Subnetwork A. Sampling bias could be a possible explanation. Alternatively, this uneven distribution of Atlantic haplotypes may reflect historical migration patterns, where ancestral sea beet populations expanded from their origin in the Mediterranean region to the Atlantic coast, during which mitochondrial diversification occurred. The dissemination of sea beet from the Mediterranean to the Atlantic is now widely accepted [20,37]. However, several Mediterranean haplotypes are found within lineages dominated by Atlantic haplotypes (e. g., Haplotype 28; Fig 2B), and vice versa. This pattern suggests a more complex evolutionary history potentially involving reverse (Atlantic-to-Mediterranean) seed movement after haplotype divergence. A comprehensive study of sea beet mitochondrial diversity will be essential to fully understand these dynamics.

Mitotypes polymorphism is nearly equivalent between the Mediterranean and Atlantic regions with diversity indices of 0.8733 and 0.8725, respectively. However, the presence of region-specific mitotypes and differences in the frequencies of shared mitotypes result in statistically significant genic differentiation between the two populations. Within each region, mitotype polymorphism across subregions ranges from 0.6930 to 0.8424, indicating relatively high diversity. These findings suggest that mitochondrial polymorphism is broadly high across sea beet populations at a macro-geographic scale, with the notable exception of DNK, which exhibits markedly reduced diversity.

We observed a striking reduction in mitochondrial polymorphism among the DNK accessions. This reduction is consistently evident across the two datasets: all DNK accessions possess Haplotype 1 in the SNP dataset and min07 in the minisatellite dataset. Although the geographic sampling range in DNK is narrower compared to other regions (Fig 1), the complete uniformity of mitotype—only one detected across 11 DNK accessions—stands in contrast to the neighboring subregion, Atl:FRA-GBR, where the average number of mitotypes is 1.80 per accession. Note that a Swedish accession BETA 1960, collected near DNK, also possessed Haplotype 1 (S4 Table).

DNK represents the northernmost extend of the sea beet’s range [20], and is therefore likely the youngest population. Haplotype 1 is also found along the Atlantic coast of France, the United Kingdom and Ireland (S4 Table), and the frequency of min07 is high in Atl:FRA-GBR. Thus, the occurrence of Haplotype 1 and min07 in DNK is unsurprising. However, other high-frequency mitotypes in Atl:FRA-GBR, such as min09, are absent in DNK. This suggests that all mitotypes initially migrated into DNK but min07 became extinct. Since then, no additional mitotypes have been successfully introduced to, or persisted in, the DNK population. A possible explanation for the mitochondrial monomorphism observed in DNK is a combination of a small number of the initial migrants and restricted gene flow (i.e., founder effect). In this case, the nuclear DNA polymorphism in the DNK population would be much lower than in other populations, and this demographic history would be detectable in their genomes. This warrants further investigations in future studies.

A less likely, but attractive, possibility is that the absence of CMS is associated with this pattern. As neither Haplotype 1 nor min07 is associated with CMS, the DNK population appears to be free from CMS mitochondria. Another subregion where no CMS-associated mitotypes were detected is Med:TUR-CYP-ISR-EGY. However, in contrast to DNK, Med:TUR-CYP-ISR-EGY shares nine mitotypes with the neighboring Med:GRC-TUR subregion, including rare types such as min33 and min35 (Table 2). This suggests that mitochondrial migration and survival may be more feasible in the Med:TUR-CYP-ISR-EGY region. It cannot be ruled out that Med:TUR-CYP-ISR-EGY was previously invaded by CMS mitochondria, especially given their presence in Med:GRC-TUR and other Mediterranean subregions (Table 4).

A theoretical prediction suggests that the presence of CMS plants within a population may lead to increased mitochondrial polymorphism [7]. CMS mitochondria have been found in both Atlantic and Mediterranean sea beet populations ([18,22] and this study), which, with the exception of DNK, exhibit high mitochondrial polymorphism. In contrast, DNK —a CMS-free subregion—harbors a single mitotype. Thus, the case of sea beet suggests a correlation between CMS and elevated mitochondrial polymorphism, which could serve as a potential index of CMS detection within populations. While this apparent correlation between the presence of CMS and elevated mitochondrial polymorphism is intriguing, we believe that the absence of CMS alone cannot fully explain the reduced polymorphism observed in DNK. Further research, both theoretical and empirical, will be necessary to elucidate whether and how CMS mitochondria influence mitochondrial diversity in natural populations.

## Supporting information

S1 Fig Haplotype network of sea beet mitochondria. Haplotype network of sea beet mitochondria. Circles represent mitochondrial haplotypes. Dashed lines show alternatives. Branch length is proportional to the number of mutations between the haplotypes except for those among Haplotypes 34, 50, 56, 38 and 46 as these haplotypes are too similar to each other to draw correct network, and except for the branch between Haplotypes 44 and 12 as the number of mutations is too large to draw the branch in proportional scale. Alternative branches between Haplotypes of 1, 7, 9, 11, 18, 19, 39, 40, and 57 are not shown. The original network is split between Haplotypes 25 and 4 (denoted by triangles) into Subnetwork A (panel A) and Subnetwork B (panel B). Accessions with the haplotypes are summarized in S4 Table. Colors of circles indicate the collection sites of the accessions with the haplotype: red, Mediterranean area; sky blue, Atlantic coast; and white, both. Haplotypes with accessions having Owen, G, or E CMS are shown by green-highlighted labels.
(PPTX)

S1 Appendix Nucleotide sequences of 270 sea beets reconstructed 749 mitochondrial SNPs sites. (TXT)

S1 Table Sea beet accessions and mitotypes. (XLSX)

S2 Table Identified mitotypes and their allelic constitution. (XLSX)

S3 Table Sea beet accessions in haplotype network. (XLSX)

S4 Table Mitotype and number of repeat units in minisatellite loci. (XLSX)
