# Supplementary figures and images for: Mitochondrial polymorphism of sea beet (Beta vulgaris ssp. maritima), a species with cytoplasmic male sterility
Source: PLoS One. 2025 Sep 23;20(9):e0332940. doi: 10.1371/journal.pone.0332940 (PMC12456822; doi:10.1371/journal.pone.0332940)

## Slide 1
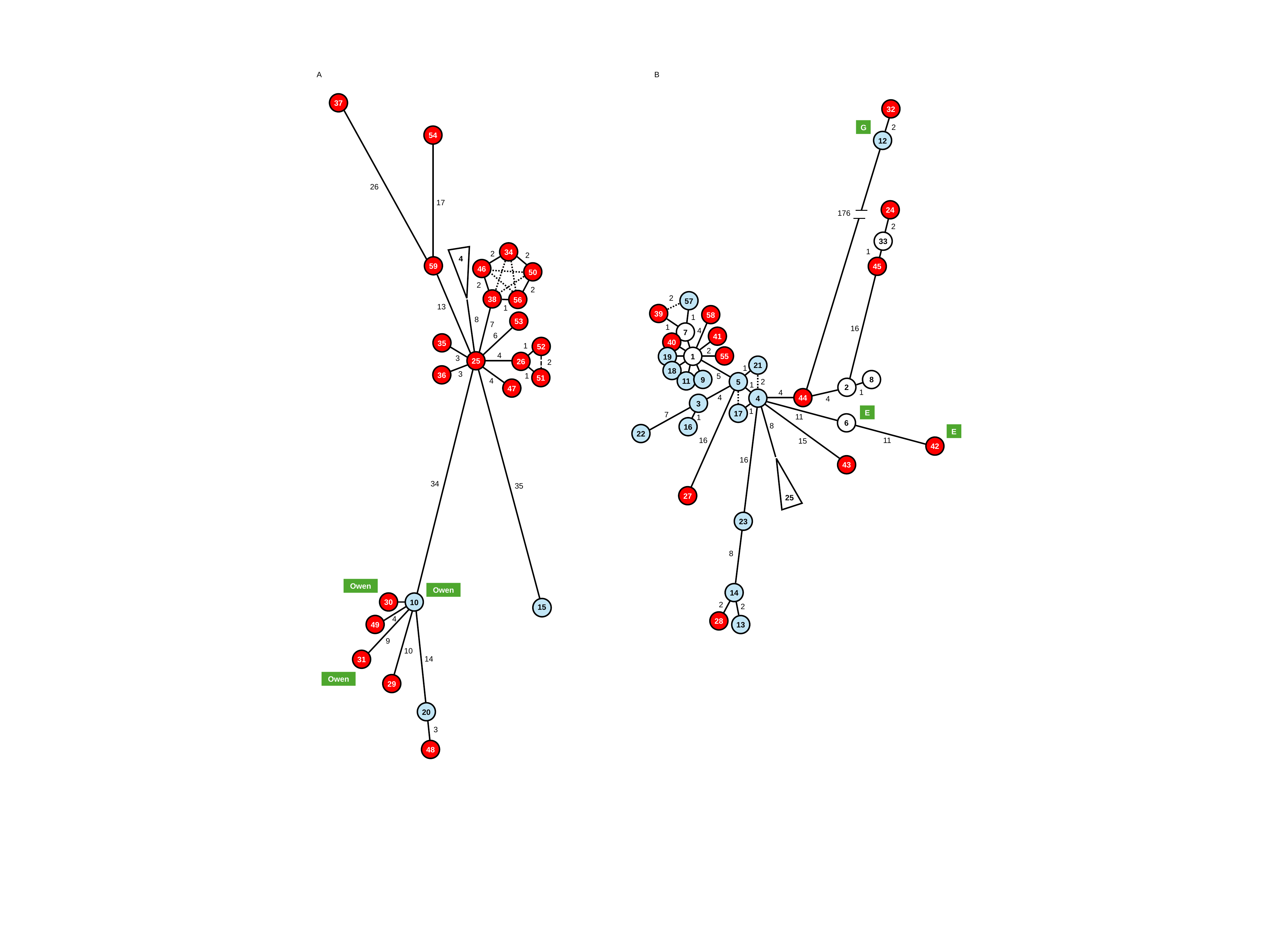

A
B
37
54
26
17
59
32
2
G
12
24
176
2
33
34
46
50
38
56
1
2
2
4
45
2
2
2
57
13
1
39
58
1
53
8
7
1
16
7
4
41
6
40
35
52
1
2
55
1
19
4
3
25
26
2
21
1
18
15
36
3
1
51
5
9
11
8
5
17
4
2
1
47
2
1
4
44
4
4
4
3
1
E
7
11
1
6
16
8
E
22
11
16
15
42
16
43
34
35
27
25
23
8
Owen
Owen
14
30
10
4
49
9
10
31
14
29
20
3
48
2
2
28
13
Owen

Supplement: S1 Fig — Haplotype network of sea beet mitochondria. Circles represent mitochondrial haplotypes. Dashed lines show alternatives. Branch length is proportional to the number of mutations between the haplotypes except for those among Haplotypes 34, 50, 56, 38 and 46 as these haplotypes are too similar to each other to draw correct network, and except for the branch between Haplotypes 44 and 12 as the number of mutations is too large to draw the branch in proportional scale. Alternative branches between Haplotypes of 1, 7, 9, 11, 18, 19, 39, 40, and 57 are not shown. The original network is split between Haplotypes 25 and 4 (denoted by triangles) into Subnetwork A (panel A) and Subnetwork B (panel B). Accessions with the haplotypes are summarized in S4 Table. Colors of circles indicate the collection sites of the accessions with the haplotype: red, Mediterranean area; sky blue, Atlantic coast; and white, both. Haplotypes with accessions having Owen, G, or E CMS are shown by green-highlighted labels. (PPTX) [file pone.0332940.s001.pptx]
